# The Hospital. Nursing Section

**Published:** 1902-02-15

**Authors:** 


### The Hospital.

### Nursing Section.

Contributions for this Section of "The Hospital" should be addressed to the Editor, "The Hospital" Nursing Section, 28 & 29 Southampton Street, Strand, London, W.C.

No. 803.—Vol. XXXI. *SATURDAY, FEBRUARY* 15, 1902.

### Notes on News from the Nursing World.

### AN OBJECT LESSON FOR THE DEPARTMENTAL COMMITTEE.

Every member of the Departmental Committee now inquiring into the nursing of the sick poor in the workhouse infirmaries will, of course, have perused the evidence given in the case of the Dudley Workhouse scandals, which is summarised in another part of the paper, in order that the decision arrived at by the Local Government Board may be clearly understood by our readers. We do not suppose that the Dudley Board of Guardians are below the average in either capacity or judgment, and their treatment of a trained superintendent nurse who knows her duty, their toleration of the extraordinary conduct of some of the untrained workhouse officials, and their evident impression that they had nothing to reproach themselves with, may be regarded as a sample of the sort of thing that is going on in the smaller workhouses all over the country. It may be admitted that Miss Newbury showed some want of tact in her attitude towards the Guardians, but the state of affairs with which she had to deal was calculated to try the patience of a Job. Her reinstatement in the position from which the Guardians wrongfully deposed her, and the reprimand administered by the Local Government Board to the workhouse master and his subordinates, are the best proof that could be afforded of the excellence of her work and of her fitness for the post she holds.

### THE NURSES' CO-OPERATION.

The voting at the meeting on February 6th to fill the vacancies on the Committee of the Nurses' Co-operation was as follows :—Nurse Janet Christie, 188 ; Nurse Janet Rogers, 95 ; Nurse H. Green, 93 ; Nurse E. Turner, 69. To succeed as the representatives of the whole body of nurses in the organisation it is essential that the four elected members should always act and vote together, and that they should keep their fellow-members well informed of what is going on, so as to promote everybody's interest and well-being within the Co-operation.

### GUY'S HOSPITAL.

We are informed that, in connection with the Nurses' Recreation Society, a League of Past and Present Nurses has recently been established at Guy's Hospital. The matron will therefore be glad to receive the name and address of every nurse trained at Guy's, in order that the aim and objects of the league may be brought under her notice.

### NURSING IN AFGHANISTAN.

According to Dr. Lillias Hamilton, than whom there is no higher authority, there are practically no nursing arrangements in Afghanistan. Miss Hamilton's former nurse, Mrs. Daly, is still the only member of her profession in Cabul. We are glad to learn that her ministrations are much prized by the inhabitants of the town and neighbourhood, but she must be greatly in need of assistance.

### THE HIGHER EVOLUTION OF NURSING.

In the course of a series of "Suggestions for the Improvement of Training Schools for Nurses," Dr. Cabot, physician to Massachusetts General Hospital, lays down four propositions : That nurses should pay for their training and be taught by paid instructors ; that nursing should be taught by nurses, medicine by physicians ; that nurses should be prepared for private nursing by practice in families outside of a hospital, and by the teaching of nurses in active practice ; and that the nurses' training should not be exclusively technical, but should include some liberal studies, such as sociology, history, and literature. As to the first, we go so far with Dr. Cabot as to admit that it might tend to bring into the profession a better educated class of women, but we are satisfied that, in this country at any rate, the payment by nurses for their training would have a disastrous effect in rendering it necessary for private nurses to charge fees which the overwhelming majority of the public could not pay. The advantage of nurses teaching nursing are beyond doubt, and we have always urged the importance of hospital training being supplemented by experience in private nursing. Concerning the fourth proposition, Dr. Cabot truly says that the nurse's life, both before and after training, is so confined and so isolated that she is especially in need of the inner resources which liberal education fosters. All that she can learn not only from sociology, history and literature, but also from art and the drama, must be a help to her, provided, of course, that the teaching is good. Fortify and inspire her, by all means, with the consciousness "of the more normal and successful sides of life" ; but it is possible to overdo even culture, and it must never be forgotten that the vital point in nursing is to be able to concentrate attention on the duty of the moment, however severely technical.

### LEEDS NURSES AND THE NATIONAL PENSION FUND.

At the twenty-sixth annual meeting of the Leeds Trained Nurses' Institution it was stated that the staff now numbers 91 engaged in private nursing, and 18 probationers in training at hospitals. During last year 14 nurses left, two in order to be married, three were giving up nursing, one has undertaken mission work in Central Africa, and the remainder have accepted engagements elsewhere. It transpired that the sum of £200 out of the receipts for nursing had been paid over to the Leeds Trained Nurses' Institution Trust Fund of the Royal National Pension Fund for Nurses, several of the nurses being now recipients of that fund.

### ST. PATRICK'S NURSES.

At the annual meeting of the supporters of the St. Patrick's Nursing Home, Dublin, it was stated that the number of visits paid by the nurses to the sick poor had reached the great total of 37,768 during last year. One of the speakers alluded to the difficulty experienced in obtaining subscriptions from the large employers of labour in the city, although no class benefited more from the work, because it is of paramount importance to them that when their employees are incapacitated they should be restored to health as quickly as possible. Unfortunately, for the first time during the 25 years of the existence of the institution, the report showed a deficit of £65, although the poor themselves contributed over £30 in small subscriptions. But towards the close of the meeting the friends of the Home were cheered by the good news that the directors of Guinness' Brewery had decided to contribute £100 this year instead of £60 as usual. It is interesting to notice in the report that when Queen Alexandra received Queen Victoria's nurses at Marlborough House last July, St. Patrick's Home was represented by the superintendent, the assistant superintendent, and seven staff nurses, their expenses being defrayed by the members of the committee.

### BOGUS NURSING INSTITUTIONS.

It is with much satisfaction that we note the conviction and punishment of a woman pretending to carry on nursing institutions at Manchester. This person, a widow named Bishop, who appeared in the dock dressed as a nurse, had been conducting establishments, described at Manchester, as "The British Israel, Anglo-Israel, Hebrew Sisters," and at Leeds, as "The Ten Sisters of the Home of Israel, Nursing Sisters to the Poor, originated in honour of the 80th birthday of Her Majesty Queen Victoria." She advertised for girls under 14 years of age, and for orphans; and so credulous are the public, or so willing to shift their responsibilities to other people, that she obtained the charge of a large number of children. Towards three of these she was specifically accused of acts of gross cruelty, and the evidence being of the most conclusive character, she was sentenced by the bench to three months' imprisonment with hard labour. One of the victims of cruelty, a girl of 15, named Florence Harley, said, she went to the Leeds home two years ago, and understood that she was to be trained as a nurse. Of course, she did not receive any such training, but she was dressed in a black and white cloak, and a black bonnet, and on her arm she had a red cross. That a child of this age should be imposed upon is not surprising, but that the father of the two other children, aged respectively eight and four, should, as he stated, have handed them over to Mrs. Bishop in the faith of her assurance that they would be trained "in hospital work," is amazing. The woman, in her defence, did not attempt to show that she had ever received any kind of training as a nurse, and the bogus nursing institutions out of which she seems to have made money have now received their quietus.

### PERSONALITIES AT LONDONDERRY.

There was a curious passage-of-arms at the meeting of the Londonderry Board of Guardians when the question of the election of nurses was under consideration. Dr. Alexander moved that Nurse Hurley be appointed fever hospital nurse "as she had better testimonials than the other." Thereupon Mr. Gregg inquired, "What age is she?" Dr. Alexander promptly rejoined, "She is younger than you. She is only 26." This sally was received with loud laughter which was turned against the speaker when another Guardian interposed with the remark, "She is maybe the handsomer of the two." Mr. Gregg rejoined that he did not think being two years younger was a recommendation for a fever hospital nurse, and the majority of the Guardians evidently considered that it was no advantage, for they elected the senior candidate by 23 votes to 15. It is quite conceivable that Dr. Alexander's introduction of pleasantries did not benefit the nurse whose claims he advocated.

### BARNSTAPLE WORKHOUSE INFIRMARY.

At the last meeting of the Barnstaple Guardians Mr. Preston Thomas, of the Local Government Board, pressed home the fact that now the children have been got out of the workhouse into homes, the Guardians have an opportunity of making better provision for the sick. He said he was quite sure that among the things which would commend themselves to the Guardians was some addition to the nursing staff. For a considerable time the provisions of the order for the nursing of paupers had, he pointed out, been violated in that workhouse, and on the male side to a large extent the sick had been left to the care of other paupers. He thought that the rooms vacated by the children would make excellent sick wards if money were spent on them. Mr. Thomas added that if the Guardians wanted to see how old-fashioned wards could be turned into good wards, meeting the requirements of the present day, they need not go farther than Okehampton, which from being one of the worst workhouses in the district had become one of the best. The chairman told the inspector that the nursing question had been "settled," but he did not state in what way.

### AN IRISH SCANDAL.

A communication has been received by the Board of Guardians of the Kanturk Union from the Local Government Board for Ireland with regard to the report of their medical inspector, Dr. E. C. Bigger, relative to the charges made by a fever patient in the workhouse hospital against Miss Lena Lineham, nurse, and Miss Hannah Fitzgerald, attendant. Inquiry had also been instituted into the general management of the workhouse hospitals. The patient, Charles Brodrick, charged Miss Fitzgerald with skimming the patients' milk; that on one occasion she refused to get him a poultice from 12 o'clock noon till 8 p.m.; that he was put into a bed after the discharge of another patient without the bedclothes being changed, and with a portion of a poultice sticking to the blankets, etc. The Local Government Board commented in their report upon the unsatisfactory circumstance that it was nearly two years after the occurrence of the matters complained of before the complaint was brought forward. Also, the patient referred to had proved himself an unsatisfactory witness in other matters. They considered the charge of skimming the milk not proved, but believed there was foundation for the other charges. Evidence was forthcoming at the inquiry that there were 44 male and 42 female patients under treatment in the two hospitals, in charge of two nurses, two assistant nurses, two night nurses, and two paid attendants for the children. One of these nurses had received midwifery training in the Rotunda Hospital, another three months' midwifery training in the Lying-in Hospital, Cork, and none of the remainder any training whatever. Mention was made of the fact that Dr. Bigger informed the board that the nurses appeared to have no system in doing their work, nor did they seem sufficiently careful of their dressings, instruments, etc. "There seemed to be no regulations as to nurses' duties, hours for meals or recreation, and no written reports were kept by the day nurses." The Local Government Board trusted that the guardians would not hesitate to appoint a trained nurse at once, as the services of such a nurse would add considerably to the order, system, cleanliness, and comfort of the patients, and reminded the guardians that half the salary of a fully-trained nurse would be recouped from the Local Taxation Account. The guardians deferred the consideration of this important communication till the next board day.

### MORE WORK WANTED.

At the fourth annual meeting of the Shrewsbury Victoria District Nursing Association the committee drew attention to the fact that not only were further subscriptions required, but a more extended interest in the association was greatly to be desired. The expenditure was £220; the income £219, including £50 grant from St. Chad's Charities Trustees, and if their yearly contributions were withdrawn at any time matters would look very serious. Also it was stated in the report that the nurses in the past had not found full employment for their time, and that many cases of suffering which might have been relieved by their attention had not been brought under their notice. The total number of cases nursed was 152, and the number of visits paid to patients 3,918. The Mayor of Shrewsbury was appointed president at the meeting, and he took the opportunity of stating that the mayoress was instituting a movement in the town for collecting money for the parent institution, that it was being very heartily taken up, and that his wife had received a large number of promises. It had been suggested that some portion of the proceeds of the collection should be devoted to the local association, and the mayor thought the suggestion worthy of serious consideration, although the London authorities did not quite agree with the idea. The question why the Nursing Association had not proved more successful was discussed at length, and one of the nurses sagaciously suggested that many of the poor people imagined that they had to pay for attendance. The matter was left in the hands of the committee to consider how best to draw the attention of the poor to the real nature of the work of the association.

### A NURSES' HOME FOR TUNBRIDGE WELLS.

At the ninth annual meeting of the Tunbridge Wells District Nursing Association, the secretary stated that the past year had been marked for the Association by an increase of work, necessitating first the temporary employment of a nurse from Westminster Hospital during the illness of one of the staff; then for ten weeks during the holidays the services one of the Kent nurses were required; and lastly, the addition of another probationer to the staff, the expenditure involved being partly justified by the expectation of a permanent free Home for the Nurses promised as the local memorial to Queen Victoria. In the year of the Diamond Jubilee the President of the Nursing Association strongly advocated making such a Home the Jubilee Memorial. The proposal did not at the time find general favour, but during the past year, with the help of lady collectors and owing to the work of several influential persons, £600 had been secured. The Mayor subsequently appealed to the public with the result that £2,000 is now in hand. It is hoped that the Home will be ready for occupation during the course of the present year. The saving of £45 a year, hitherto spent for rent, will help towards the additional expense of the fourth nurse. The increase of work has, according to the report, been due in great measure to the growing popularity of the nurses. The poor have shown their appreciation by subscribing themselves—in addition to £17 for the Memorial Home—a much larger sum to the Association than on any previous occasion. During 1901 the nurses had nursed 75 more cases than in the previous year, and paid altogether 10,272 visits.

### SOUVENIR OF THE PENSION FUND FETE.

We are asked to state that there are only a few copies of the Souvenir of the fete at Marlborough House last year which were specially reserved for the nurses of the Royal National Pension Fund left. Those who desire to possess either the album or the plate, or both, should therefore apply at once to the publishers of The Hospital, 28 and 29 Southampton Street, Strand, W.C.

### KIMBERLEY NURSING ASSOCIATION.

At the annual meeting of the Kimberley Nursing Association, the employment of a second nurse was suggested, and as the population of the districts of Kimberley, Nuttall, Watnall, Strelby, and Bilborough exceeds 7,000, it can hardly be said that one is sufficient. Last year the single nurse attended 108 cases and paid nearly 2,000 visits. The financial state of the association is satisfactory; but as it appears that several of the local organisations are about to amalgamate, the question of providing her with a colleague was not proceeded with.

### SHORT ITEMS.

The Prince and Princess of Wales, on the occasion of their visit to St. George's Hospital last Friday, were shown over the nurses' new quarters.—The Hon. Mrs. Evelyn Hubbard has handed to the committee of the Women's Memorial to Queen Victoria, in connection with the Queen's Nurses, the sum of £1,000, as an instalment of the fund now being raised by the Mothers' Union for that purpose. The amount includes the mites of poor women in all parts of the country.—The annual general meeting of the Hammersmith and Fulham District Nursing Association will take place next Wednesday afternoon at three o'clock, at St. Andrew's Hall, West Kensington, the Mayor of Fulham in the Chair.—The third sessional lecture in connection with the Royal British Nurses' Association will be delivered at 10 Orchard Street, W., by Dr. Robert Bowles, on Thursday, February 20th, at 5.30 P.M. The subject will be "The Importance of Position in Accident, Disease, and Unconscious Conditions, such as Apoplexy, Anæsthesia, Drowning, etc."

### Lectures on Gynæcology for Nurses.

By Robert Jardine, M.D., M.R.C.S., F.F.P. and S.G., F.R.S.E., Senior Physician to the Glasgow Maternity Hospital Examiner in Midwifery to the University of Glasgow.

### LECTURE V.—AMENORRHŒA.

By this term is meant the absence of menstruation when it should naturally exist. Amenorrhœa is, of course, normal in a young girl before puberty, and in women after the menopause, as it also is during pregnancy and lactation. This may be known as physiological amenorrhœa. In exceptional cases menstruation occurs in very young girls, and also sometimes during pregnancy and lactation. In women beyond the menopause hœmorrage occasionally occurs, but this will be found to be due to disease, generally cancer, and it is therefore not true menstruation.

*Pathological Amenorrhœa.*—Menstruation is in abeyance either from some cause connected with the pelvic organs or with the general condition of the patient.

*Causes Connected with the Pelvic Organs.*—In some cases the uterus and ovaries are entirely wanting or are only rudimentary. This is due to maldevelopment, and manifestly menstruation cannot occur when these organs are wanting or very rudimentary.

Absence or occlusion of the vagina, as from an imperforate hymen, or the result of cicatricial contraction of that passage from wounds or ulcers, or occlusion of the cervical canal from an injury. As a matter of fact this is not true amenorrhœa, because if the uterus and ovaries are present the process will be going on; but, as the external passage is closed, the discharge will be accumulating behind the obstruction. This gives rise to a very serious condition.

Diseased conditions of the uterus and ovaries, *e.g.* atrophy of these organs after an attack of inflammation, or from adhesions binding them down after pelvic peritonitis; cystic disease of the ovaries, or when the ovaries and tubes or uterus have been entirely removed.

*Causes Connected with the General Condition of the Patient.* From abnormal conditions of the blood in connection with constitutional diseases such as fevers, chlorosis, phthisis, cirrhosis, or Bright's disease of the kidneys, and from severe hæmorrhage, as from piles or a wound. It is sometimes caused by abnormal states of the nervous system, as in mental depression, hysteria, etc. Fear may cause it, as when a woman fears that she is pregnant. A severe shock or disappointment in love may act in the same way. Overstudy among schoolgirls is not an uncommon cause. Change of mode of life may also cause it, as may often be seen among nurses shortly after beginning their training. It is sometimes seen in indolent, luxurious women who live unnatural lives and take very little exercise in the open air. Sometimes the cause may be a chill just before the period is due, or a period which has commenced may be checked in this way.

We may class amenorrhœa as primary where the cause is a defect in the genital organs, and the menses have never appeared at all, and secondary when they have appeared, but are suppressed for a time. At the commencement of the ending of the menstrual life, as we have already mentioned, there may be considerable irregularity, so that at these stages of life there may be periods of amenorrhœa.

*Diagnosis.*—From the general appearance of the patient one can often form a shrewd guess as to the cause. A careful history of the patient's previous health must be got. The question of pregnancy must always be borne in mind. If a young woman has been perfectly regular prior to the suppression and seems in good health, the chances are that the amenorrhœa is either physiological, *i.e.* due to pregnancy, or it has been caused by fear. Judicious questioning will generally lead to a discovery of the truth.

If the condition is manifestly due to constitutional disease' such as phthisis, chlorosis, &c., a local examination will not be necessary. When there is a suspicion of pregnancy the breast condition is of importance, and medical men can usually get an opportunity of examining these parts when auscultating the heart and chest. If the areola around the nipples is very large and darkened, it gives one evidence of pregnancy. In such a case morning sickness should also be inquired after. In unmarried girls a local examination will not be made unless there are good reasons for doing so; but this will be a question for the doctor to decide.

*Treatment.*—Of course nurses have nothing to do with ordering treatment; still, in a matter like this, in which nurses are very likely to be told about the condition, even before the patient is taken to a doctor, it is well that they should know the directions which treatment may not impossibly take, and the first thing to remember is that where there is any suspicion of pregnancy no attempt must be made to restore what the patient may regard as the natural condition of affairs. Any interference with the proper course of pregnancy is criminal. In other cases the treatment adopted will depend upon the view which is taken as to the cause of the abnormality. When this is of a constitutional nature the periods will probably return when the health becomes re-established; while when a sudden suppression has taken place, as from a chill, hot hip-baths or foot-baths or a large poultice over the lower part of the abdomen are useful.

When the uterus is infantile—that is, not fully developed —galvanism sometimes does good. In those cases in which the uterus and ovaries are wanting nothing can be done. Where there is an occlusion in some part of the genital canal an operation will be necessary to remove it. This must be done with the strictest aseptic precautions, and the fluid which is discharged afterwards must be allowed to drain away slowly into aseptic dressings. Sepsis is apt to occur in such a case if the greatest care is not taken.

I have not mentioned certain drugs which are sometimes used in the hope of restoring the periods. They are very uncertain in their action, and, when they act, they are full of danger. All that a nurse needs to know about them is that they should never be used by her. Judging from the large number of quack nostrums so openly advertised in the lay press it is quite evident that they are extensively used. A nurse should never lose an opportunity of dissuading women from using them.

Among women generally there seems to be a great dread of the bad effects of amenorrhœa. There is no doubt that where it arises from serious constitutional causes the condition is a grave one, not from the irregularity itself, but from the general effect of the disease which has caused it; as, for instance, in phthisis in which amenorrhœa generally results. I have frequently had a mother beg me to give her girl something to bring on the periods, as she was quite sure that the patient would die if they did not come on. In such cases, however, the condition is probably an effort of Nature to avoid loss of strength. We see the same in cases of chlorosis or profound anæmia from great loss of blood. Here the amenorrhœa is conservative. The effect must not be confounded with the cause.

### Presentations.

Royal Aberdeen Hospital for Sick Children.—Miss Katherine M. Lumsden, who is resigning the post of Hon. Superintendent of the Royal Aberdeen Hospital for Sick Children, which she has held for 16 years, has been the recipient of a handsome antique bureau from the past and present medical staff of the hospital, 12 in number. The reason for the choice of the bureau which belonged to the late Earl of Moray, and is believed to be two or three hundred years old, is because the donors were aware of Miss Lumsden's deep appreciation of antique furniture.

### Prevention of Bed-sores.

### EXAMINATION QUESTIONS FOR NURSES.

THE question for January was as follows:—What measures would you take to avoid bed-sores in the case of helpless patients?

### FIRST PRIZE.

The Measures to take to avoid Bed-sores in the Case of Helpless Patients.—The back should be washed with soap and warm water at least twice daily; also after every time a motion is passed.

It should be rubbed with some kind of spirit (methylated is most generally used, but rectified spirit, brandy, whisky, or good Eau de Cologne will do); afterwards it must be well dusted with zinc and starch powder. Great attention must also be paid to the hips, knees, heels, elbows, shoulders, and ears, which must be treated in the same way as the back.

The bottom sheet must be kept perfectly smooth and straight, and free from crumbs, etc. A draw-sheet must be stretched tightly across the bottom sheet, and drawn through at least three times a day, also whenever soiled. If the patient passes urine or motions unconsciously, a wide macintosh sheet must be placed under the draw-sheet, great care being taken that it does not get into rucks. Pads of wool and tow must be placed under the patient to catch as much of the discharge as possible. Both bottom and draw sheets must be immediately changed when soiled or wet.

The back will require some ointment rubbed over it to prevent the urine, etc., soaking into the skin. Zinc made up with vaseline is the best.

If the patient is very thin, if possible, and with the permission of the medical attendant, an air or water bed should be procured at once. The nurse should see that it is not filled *too* full, as it then becomes hard and uncomfortable. Failing an entire bed, a half-bed will do, reaching from the shoulders to the thighs. The heels may be prevented from rubbing the bed by placing pads of wool under the ankle.

In the majority of cases a small air or water cushion is not of much use, as the patient so easily slips off it.

Care must be taken when giving the bed-pan or slipper not to bruise or scrape the back.

It is best for the patient only to wear a single short shirt or night-gown, not to reach much below the waist, so that it cannot get into creases under the back. If the patient is quite unconscious, an ordinary night-shirt, split right down the front, and put on back to front, is the easiest to adjust, and disturbs the patient the least; change of position is also a great help. Do not let the patient lie on his back for long together if it can be avoided, but turn him every hour or so, if possible, from side to side, if necessary propping him up with a pillow.

There are very few cases indeed where, with sufficient care, a bed-sore cannot be avoided. FRANCES.

### SECOND PRIZE.

The first thing to consider in the prevention of bed-sores is, the bed. A spring bedstead and hair mattress are the best, and a helpless patient should be placed, if possible, on a water-bed or a large water-pillow. Great care must be taken to keep the under-sheet, draw-sheet, and macintosh free from rucks; the macintosh may be fastened with safety-pins. The patient should not lie on the nightdress (which will be opened up the back), as it is very liable to ruck.

The bed must be kept free from crumbs, and soiled sheets changed immediately. The draw-sheet should be a good width, put on with the hem at the bottom, and free from patches, darns, and seams.

Ring pads should be applied wherever there is extra pressure, especially to prominent bones; a pad for the ear will often be a great comfort to a patient obliged to lie on one side. Pads should always be put under the heels of patients who cannot be turned on the side, as bed-sores are likely to occur before one is aware.

Bed-sores are most likely to come on the shoulders, elbows, sacrum, hips, and heels. In addition to the regular washing with soap and water, these parts should be well rubbed with methylated spirit and starch-powder at least twice a day. It is important to remember that rubbing is in itself of great value in keeping the skin in good order. Zinc ointment and zinc powder are better in cases of great emaciation, or if the patient complains of soreness. Hazeline and lanoline ointment is a good protective against damp, in cases where there is loss of control, and olive oil seems to relieve the tension of the skin in cases of dropsy. But, whatever dressings are chosen, scrupulous care and regularity in their use will be found the best preventative. Some patients seem peculiarly liable to contract bed-sores, and they are more likely to occur in diseases which cause great emaciation, as phthisis, and in those which interfere with the nutrition of the skin, as enteric fever, dropsy, and paralysis. Such cases need special care and watching, and should be attended to as often as an opportunity can be made without disturbing the patient too much.

If, in spite of all her efforts, a bed-sore seems inevitable, the nurse should inform the doctor, and not wait till it is well-established before letting him know.

NANFANS.

### LARGE NUMBER OF COMPETITORS.

I have received 411 answers to the question, and the average is good. The chief fault is that most candidates dwell rather too much on the subject of the bed, and too little upon the necessity of perpetual attention and continued gentle friction to restore impeded circulation. *Pressure*, as most competitors rightly say, is the cause of bed-sores—but *why?* Because pressure impedes circulation, the tissues are not nourished, they become dead, and a sore quickly forms—therefore our main object must be to prevent pressure as much as possible, and, when not preventable, to restore circulation by means of friction. As to the vehicles used, spirit is good and necessary, though not suitable to all skins, as some of the writers notice; but dusting powder, which almost all advocate, is of doubtful utility for this reason—there is always more or less perspiration, often to a serious extent; this, uniting with the powder, forms an unwholesome paste, most deleterious under the circumstances. As friction should be kept up at least for five minutes each time that the patient is attended to, it is advisable to use some ointment *always* as a lubricant to avoid the dragging sensation inseparable from rubbing with spirit alone.

### HONORABLE MENTION.

As the number of candidates is so large this month, six honorable mentions have been awarded to "A. H. R." (whose paper is so good that she nearly won the first prize), to "Val," "Anin," "Nurse E. W." (to Pseudonym sent), "Anglican," and "Phenacetin."

### QUESTION FOR FEBRUARY.

How would you treat bed-sores in their various stages if the medical man left such treatment to your discretion?

### CAUTION.

If the doctor orders treatment for bed-sores you will, of course, implicitly follow his orders, but very frequently he leaves it to the nurse to try various remedies, and only overlooks her efforts. In district nursing appalling specimens of bed-sores are met with, and bed and bedding are generally far from desirable; a nurse's ingenuity is taxed to the uttermost to relieve and, if possible, remove the scourge. Frequent change of dressings is advisable; very often one drug seems to lose all power, then another must be tried, until that too loses its usefulness, and so on, with never-varying assiduity till improvement is assured.

THE EXAMINER.

### RULES.

The competition is open to all. Answers must not exceed 500 words, and be written on one side of the paper only. The pseudonym, as well as the proper name and address, must be written on the *same* paper, and *not on a separate sheet*. Papers may be sent in for fifteen days only from the day of the publication of the question. All illustrations strictly prohibited. Failure to comply with these rules will disqualify the candidate for competition. Prizes will be awarded for the two best answers. Papers to be sent to "The Editor," with "Examination" written on the left-hand corner of the envelope.

N.B.—The decision of the examiners is final, and no correspondence on the subject can be entertained.

In addition to two prizes honourable mention cards will be awarded to those who have sent in exceptionally good papers.

### Nursing at the Dudley Workhouse Infirmary.

VINDICATION OF THE SUPERINTENDENT NURSE.

The decision of the Local Government Board consequent on the report of their Inspector and Dr. Fuller, who spent nearly a fortnight in investigating the charges brought by the Dudley Board of Guardians against Miss M. M. B. Newbury, the superintendent nurse at the workhouse infirmary, whom they had suspended from her duties, and Miss Newbury's counter charges against the officials of the Dudley Workhouse has been awaited with great and general interest in the nursing world. The decision was read at the weekly meeting of the Dudley Board of Guardians last Friday, and was to the following effect:—

The Board have carefully considered the facts disclosed by the inquiry, and they are of opinion that the superintendent nurse was very unwise in the course she adopted in answer to the Guardians' inquiries, but that the evidence seems to show that she acted in no spirit of defiance or insubordination towards the Guardians. As regards the other charges, the Board have failed to find evidence of any acts on her part that would call for so severe a course on their part as to require her resignation. On the other hand, the administration at the workhouse seems to have been open to criticism in most particulars, and frequent causes for friction seem to have arisen from the conduct of the master. The Board are not anxious to go into the many causes of complaint that are said to have arisen, although some of them are important. They may instance the fact of the master having required the attendance of the nurse on what should have been his daily visit to the sick wards. They consider that it forms no part of the duty of the nurse to attend on him during such visits unless some special cause for such attendance has arisen. The conclusion the Board have come to is that the several officers concerned should be warned that the disclosures which have been made at the inquiry have been noted against them, and will be taken into serious account should future cause of complaint arise. Meanwhile, the superintendent nurse should be allowed to resume her duties, and all the officers should be informed that, if they wish to retain their posts, they must be careful not to give needless cause to prevent the administration of the workhouse being harmoniously carried on.

### The Guardians Accept the Inevitable.

The Chairman then suggested that the report should be discussed in committee, and the following resolution was adopted:—"That the Local Government Board, having declined to accede to the request of the Guardians to dismiss Miss Newbury, she be instructed to resume her duties forthwith." In order that our readers may be able to learn the grounds of the decision arrived at by the Local Government Board, we have prepared a summary which, though necessarily greatly condensed, gives a fair impression of the principal facts:

---

### The Guardians' Charges.

The Guardians complained of the superintendent (1) that there was a want of capacity to manage those under her control, which was evidenced by the charges made by several of the nurses, as set out in the Workhouse Visiting Committee's report, dated September 24th last, and by the constant friction; (2) that it was evident that the superintendent nurse was unable to accept the position of being subject to the control of the master and matron in all matters other than treatment of the sick, as evidenced by the complaints of the master of her insubordinate behaviour both to himself and the matron, which weakened their authority and tended to disorder.

### Miss Newbury's Charges.

The charges brought against certain officers of the workhouse by Miss Newbury were as follows:—Against Nurse Ray—drinking spirits whilst on duty; drinking spirits in bedroom either whilst on duty or just before going on duty; being absent from the wards when on duty; being absent from the infirmary without leave; slapping the babies in the infirmary, and insubordination. Against Nurse Frisby—drinking spirits in bedroom either when on duty or in dinner hour just before going on duty; neglect of duty towards patients; gross incompetency, and ill-treatment of patients. Against Male Nurse Webb—insubordination and gross insolence; neglect of duty; being absent from the wards when on duty, and continued friction with female nurses. Against Female Nurse Webb—neglect of duty; leaving wards when on duty, and ill-treatment of patients. Against Mr. Walton (Master)—ignoring her authority as to matters relating to the infirmary and her nurses; interfering or allowing his wife to interfere with the nursing; interfering with her duties; failure to supply coal to the infirmary; neglect to supply various things necessary for the patients and ordered by the medical officer; supplying improper food to the infirmary wards; sending, or allowing patients to be sent up from the house to the infirmary in a filthy condition; and general annoyance both to herself and female nurses. Against Mrs. Walton (Matron)—neglect to supply of linen to the infirmary; serving out the same twice; sending up patients from the house to the infirmary in a filthy condition; interfering with the nursing; interfering with her duties and authority in the infirmary; and general annoyance to herself and female nurses.

### Appointment of Miss Newbury.

The late superintendent of the Dudley Workhouse Infirmary, Miss Peers, having volunteered for service in South Africa, Miss Newbury was appointed temporarily to fill her place on February 10th, 1900. On July 5th she was permanently appointed superintendent nurse at a salary of £40 per annum, with rations, furnished apartments, etc., rising at the rate of £1 per annum to a maximum of £45. As friction occurred from time to time between Miss Newbury and the officials, a set of rules, was drawn up and printed defining her duties. Notwithstanding this the Guardians complained that she persistently declined to confer with the master or to address him or the matron by their official titles. On September 18th Nurse Frisby and probationer Nurse Ray sent in their resignations. On September 24th the Visiting Committee held a meeting to investigate the matter, when Nurse Ray stated that Miss Newbury made her life so unpleasant she would run away rather than stop, and Nurse Frisby complained that the superintendent insisted upon her doing day duty two months and then night duty two months, instead of altering the duty every month as was arranged at her appointment. Then the chairman and members of the committee appealed to Miss Newbury to explain, and she declined to answer any questions unless they were put in writing. This the Guardians considered defiant and insubordinate, and on October 18th they suspended her from her duties. They adopted this course, they stated, in order to give Miss Newbury an opportunity, if she desired, of having a Local Government Boary inquiry. The Local Government Board, upon receipt of the news of Miss Newbury's suspension, had telegraphed that the board had no right to suspend Nurse Newbury.

### First Witness for the Guardians.

The first witness examined was the chairman of the Visiting Committee, who maintained that the attitude of the committee was quite friendly to Miss Newbury till she persistently refused to give information. Other guardians who had been present at the meeting also expressed indignation that Miss Newbury had refused to answer questions except in writing.

### The Nurses' Evidence.

Nurse Amy Ray, who was called next, said that she was engaged as a probationer in the maternity department.

Miss Newbury had, however, never given her any instructions in respect of maternity work. Neither the medical officer, nor the master, had ever complained of witness, but in consequence of Miss Newbury's attitude to her she sent in her resignation. She admitted having received medicine bottles through the post containing whisky. But the Superintendent had never spoken to her about it. The Local Government Inspector, Mr. Wethered, added after this witness's evidence that particulars of the charges of not receiving proper instruction from Miss Newbury had never been furnished either to the Superintendent Nurse or to the Local Government Board. This he characterised as "an important omission." The Solicitor to the Guardians said that it had been purely accidental.

Nurse Frisby stated that she had been badly treated by Nurse Newbury. On one occasion the superintendent threw the report book across the table, and on another told witness she ought not to be a nurse. She had found her work difficult owing to the persistent cruelty of Miss Newbury. Asked if Miss Newbury had ever found fault with her, she said that the superintendent had never done anything else. To her knowledge, she had never given any cause. Witness said Nurse Newbury had called her "silly." She allowed that this epithet was applied to her because she stated she had been outraged in the grounds, and no such thing had happened. Male Nurse Webb stated that he had been at the workhouse for eight years, that Miss Newbury had "always been hard on him" and upon one occasion deprived him of liberty the Guardians had allowed him. He was a cousin of the matron. Female Nurse Webb, according to her statement was once feeding a patient in No. 6 ward, where a grate was being repaired, and Miss Newbury came in and said, "What's this? It is one of your sneakish tricks." On another occasion, when she asked for some linen, Miss Newbury threw it at her.

Testimony of the Medical Officer.

Dr. T. F. Higgs, the medical officer at the workhouse said that he had received complaints from Miss Newbury, more or less serious, about every nurse under her. He had investigated every case, and found most of the complaints frivolous. In answer to cross-examination, he said that he had testified last week that Miss Newbury was "highly efficient, a good disciplinarian, and a kind and considerate nurse." The only fault he found against her was that she was extremely unforgiving.

Cross-examination of the Master.

Mr. Walton, the master of the workhouse, was the next to give evidence. In March of last year, whilst Miss Newbury was acting superintendent, he had to tell her that she ought to address him as "master." She replied that that was not the custom where she had come from, and when he insisted she said that she did not think she should try. He went on to give instances of Nurse Newbury acting contrary to his orders. He admitted that he reported all these things to the guardians, and yet in the face of that they appointed Miss Newbury permanently. Miss Newbury frequently told him that he had no authority to ask her to go with him to make an inventory. With reference to the permitting of a lady visitor to remain at the workhouse all night, she declined to recognise that witness had any authority at all in the matter, and in defiance of witness' orders the visitor was kept until witness invoked the assistance of the other officials. Eventually Miss Newbury left the house at 11 p.m. with her visitor, and returned alone at 1 a.m. When he had visited the infirmary, Miss Newbury treated him with contempt. He admitted that the former superintendents had made complaints against him, but those, in his opinion, were frivolous. He produced the workhouse master's report book, but it had a good deal torn out of it, which the master stated he had done himself, and the notes he produced, sent him by Miss Newbury, had been mutilated. He admitted in cross-examination that when he asked the superintendent nurse to come and make an inventory of the female linen with him, he said nothing about the matron accompanying them. He allowed that Miss Newbury had made a complaint to the visiting committee of a shortage of linen. Further cross-examination elicited the facts that his refusal to allow Miss Newbury's visitor to stop all night was due to the superintendent's behaviour that day. He had allowed the clerk and the laundress to keep visitors all night. Miss Newbury's visitor was the daughter of the late workhouse master. Mr. Walton also stated that he had been in the maternity wards, without the sister or the matron several times.

Evidence of Miss Newbury.

Several members of the visiting committee gave evidence that they had not considered Miss Newbury's refusal to make complaints, except in writing, couched in an offensive term, and then the Superintendent Nurse herself was called She refused to state her complaints, she said, other than in writing, because the doctor had told her and the nurses that the guardians were so sick of complaints that they would not listen to them unless they were put into writing. When witness went to the infirmary she found a lack of discipline. She had had complaints from the patients of having been given wrong medicine in the night. She had complained to the medical officer that Male Nurse Webb had been insolent and neglected his duty, and that she found it difficult to perform her duty with this nurse. She complained of Nurse Reeves, who subsequently wrote a letter apologising to witness for all the trouble she had caused her. She had refused this nurse a late pass, because on a former occasion she had stayed out till one o'clock in the morning. Witness had never omitted to attend the medical officer on his infirmary round. Because of the unpleasantness with Webb she had threatened to do so, but had never done it.

Causes of Friction.

The infirmary inventory included the linen the patients were wearing, which those making the inventory required to see. She wrote declining to make the inventory along with the master, but stating she would be pleased to perform the duty with the matron. With regard to the incident of the late visitor, she had never asked the master to let her keep a visitor all night before, but it was getting late, the girl was only 18, and it was some distance to her home. Witness went home with the girl and returned late. The master was very angry on the occasion and shouted at her. The visitor had been obliged to get over the gate because the porter was not at the door, and the lodge-keeper was deaf. She had regularly filled up the report papers until the supply of forms at the office became exhausted. There was friction among the workhouse officials when witness came. Miss Newbury was altogether eight hours under examination.

Nurses' Evidence for Miss Newbury.

Nurse Blackham had served under Miss Newbury at the workhouse. She had left because of the frequent quarrels which were going on. On two occasions she was locked out and had to climb the gates. Mr. Walton was very angry with her about the matter, and called her a "cheeky young hussey." He used to address her as "Blackham." In her opinion, Mr. Walton was responsible for the friction which existed whilst she was there. Elizabeth Beeson, Stratford-on-Avon, was a nurse at the Dudley Workhouse, but left because of the constant friction amongst the officers. She got on all right with Miss Newbury. Nurse Bessie Reeves said she had been nurse at the workhouse for three and a half years. There had been considerable friction between the master and Superintendent Nurse Peers. She recollected Nurse Ray receiving whisky in medicine bottles by post, and had seen her slapping children. Mary Ann Gibbs said she had been nurse at the workhouse for 27 years. She had always addressed the present master as "Mr. Walton," and he had never asked her to call him "Master." There was friction at the workhouse before Miss Newbury came. Miss Newbury was very strict about their duties. Mary Jane Darby said she had been a nurse at the workhouse for twelve months. She had received instruction in maternity work from Miss Newbury. In her opinion, Miss Newbury was not responsible for the friction in existence. She ran away last summer because she was unhappy at her bedroom being made a drinking saloon by Nurse Ray. She had found a bottle in a coal-box half-full of spirits on one occasion.

Re-examination of Miss Newbury.

Recalled, Miss Newbury stated that she blamed the master for failing to advise her that men were coming to repair the gate in No. 6 Female Ward on September 16th. She found bricklayers at work in the ward, in which were 45 patients in bed helpless, there being no nurse present. In January, 1901, Nurse Ray was granted 13 days' leave of absence without reference to witness. On two occasions Male Nurse Webb was absent without her leave, but he said that Mr. Brooks had made it all right with the master. On December 27th and 28th, 1900, no coals were supplied to the infirmary, though snow was on the ground. Miss Newbury complained, and was told by the man who carried the coal the master had told him he was not to supply her with any. Several times she went without breakfast because she had no fire to cook it with. She also complained that the master supplied improper food. Once the patients complained of the tea, which witness found smelt strongly of carbolic. When the master was asked to supply other tea, as the patients had had no breakfast, he declined. On another occasion the patients in the male ward could not drink the beef-tea. Witness tasted it. It was acid, and smelt strongly of carbolic. The master said he could not account for it, declined to supply any more, and the patients had to have arrowroot. On one occasion an inmate of the workhouse was sent to the infirmary with two bed wounds, and another transferred from workhouse to infirmary was "thick with dirt." It had been most difficult for witness to do her work owing to the continual harassing of the master. With regard to the matron, Miss Newbury complained about the shortage of linen. As she had explained to the Board—and the Visiting Committee—patients were without nightdresses, sheets were dried before the fire in the infirmary wards, the pillow cases were used sometimes for three weeks and were quite black. In February, 1901, a patient was sent from the workhouse to the infirmary covered with body lice. Other witnesses testified to seeing the master turn down the beds.

The Master's Denials.

Mr. Walton, upon being called, emphatically denied having ever turned down the beds of female patients. He knew of no order prohibiting him from entering the maternity ward. He admitted that on one occasion the tea was poor, but had no complaint that no other was supplied. When the shortage of coal was reported he sent orders to the officer in charge that coal should be sent to the infirmary at once. Miss Newbury complained because he gave a late pass to Nurse Reeves stating that in consequence the infirmary would have been left two hours without a nurse in attendance had she not undertaken the duty herself. He went to the female ward when the matron was ill to investigate the complaint of a shortage of nightgowns, and Nurse Gibbs turned down the bedclothes a little way to show him some patients had no nightdresses on. Cross-examined, he regarded it as part of his duty to give the Superintendent Nurse notice when a patient was to be discharged from the infirmary, but he might have omitted to do so on more than one occasion. Four or five officials had resigned since Miss Newbury was suspended. The matron, Mrs. Walton, denied the truth of the allegation that she had failed to supply linen to the infirmary. The laundry, however, was not quite adequate.

The inquiry terminated on December 13.

### Royal British Nurses' Bazaar.

On behalf of the movement under the auspices of the Royal British Nurses' Association, in favour of erecting a Nurses' Settlement a two days' bazaar took place last week at the town house of Lord Brassey in Park Lane, the museum and several adjoining rooms being set apart for the purpose.

At half-past two on Thursday afternoon the Princess Christian, accompanied by Princess Victoria of Schleswig-Holstein and Lady Agneta Montague, arrived, and was received by a number of ladies and gentlemen.

A bouquet having been presented to her Royal Highness, on behalf of the sale committee, by Mrs. Dacre Craven, the party made its way through the crowd of spectators into the museum; the Princess declared the sale open, and the business of the day began.

The Princess, who wore a black dress and white satin toque with black spots, presided at her own stall, and, besides the ladies already mentioned, was assisted by Lady Barlow, Lady Duckworth, and Mrs. John Langton. The stall was furnished with leather and bric-à-brac; and when the day's accounts were handed in it was found that £80 had been realised at this stall alone. In the corner close by was the "Army Nursing Service" stall, in charge of Miss Wedgwood. Here were a number of interesting war relics and a varied collection of pottery. Miss Wedgwood had the help of Sir John and Lady Furley, whose names are so well known in the nursing world in connection with the Red Cross, and a sister in her scarlet cape was busy selling china cats, while Mrs. Macpherson, Mrs. S. Muir and Miss Blanche Trew also assisted.

The feature of the members' own stall was that every article for sale had been made by the nurses themselves. [illegible]o many things had been sent in that the stall-holders [illegible]eclared that they could have gone on "filling up for a week." Mrs. Coster, Miss Grace Gordon, Miss Parsons Miss Rawson, Mrs. Campbell-Thomson, Miss M. L. Wright Miss Waller, and Miss Scott (baskets) were in charge; and the stall was furnished with some really beautiful things. Nothing could have been more tasteful than the cushions and bedspreads, or more workmanlike than the pile of useful knitted clothing; while the wooden bowls studded with brass nails were quite unique and very decorative. The other stall-holders were Lady Church, Lady Broadbent, Lady Crickett, the Lady Agnes Cooper, Mrs. Fripp, Mrs. Calvert, Mrs. Charles P. Stokes, and Miss Quain; while the London Association of Nurses and St. John's House had each furnished a stall with articles made by the nurses, friends, and patients.

Refreshments were served in the gallery under the direction of Mrs. Latter, the President being entertained by Lord Brassey's daughter, the Hon. Mrs. Freeman Thomas.

From the gallery one looked down upon a moving throng of people, among whom peripatetic stall-assistants threaded their way, offering wares of many kinds, and "*so* cheap," to the music of a string-band under the direction of Miss Wilkinson. When seven o'clock struck, it was found that the goodly sum of £570 had been realised.

The Marchioness of Lansdowne was unfortunately not well enough to keep her engagement for Friday, and at Princess Christian's special request Lady Church declared the sale open on that afternoon. All the assisting nurses wore their uniforms, and medals were conspicuous on some white aprons; two nurses worked hard at tying-up parcels in the parcel-room, and, in spite of the fatigue a bazaar cannot fail to occasion, everyone seemed thoroughly happy. One very satisfactory point about the sale was that there were no expenses. Everything was given, nothing being sold on commission, and any articles left over will, it is hoped, be disposed of at some future time for the same object.

### Charing Cross Hospital Nursing Home.

The entertainments given at the Savoy Hotel in aid of the Nursing Home Fund for Charing Cross Hospital were brilliantly successful. They were organised by Mrs. Cecil Powney and Mrs. Penn Curzon, who, with the help of many friends, including several prominent artists, produced a programme that was fresh and original, consisting of tableaux, dances and recitations. The orchestral selections and incidental music were conducted by Mr. John A. Mertens and Mr. E. J. Margetson, respectively, and the scene of the entertainments was the ballroom, kindly lent for the occasion by the directors. The tableaux, some of which were not only "living" but "moving" also, were arranged by three associates of the Royal Academy, Mr. G. Frampton, Mr. J. M. Swan, and Mr. J. J. Shannon, while Mr. Lewis Waller, Miss Esmé Beringer, and Mrs. Cecil Powney were the elocutionists; Miss Grainger Kerr generously gave her services as vocalist, and Miss Viola Tree danced, very gracefully, the dance of Bacchante from Gounod's "Faust."

The entertainment was given four times, on Monday and Tuesday afternoon and evening, and at each performance a large number of spectators was present, including a few nurses. The flowers and decorations, the new electric light effects on the stage, the artistic grouping and beautiful dresses, and the suitable orchestral accompaniments, all contributed to the success of the whole, while the programmes were as novel as Messrs. Constable could make them with the aid of gay heraldic designs.

### The Metropolitan Asylums Board.

CYCLE ACCOMMODATION.

At a meeting of the Metropolitan Asylums Board on Saturday last, it was decided, on the recommendation of the Finance Committee, to grant a superannuation allowance, at the rate of £26 7s. 6d. per annum, to Miss E. A. Lewett, aged 54, charge-attendant at Caterham Asylum in respect of 28 completed years' service.

The Asylums Committee's report recommended that the Local Government Board be asked to abolish the offices of matron of Darenth Adult Asylum, and matron of the Darenth Schools, and to authorise the appointment of one matron of the whole Darenth Asylum. The recommendation was adopted. The same committee reported that the storage accommodation for the cycles belonging to members of the staff was much needed at Leavesden Asylum; the present arrangement by which the staff keep and clean their machines in their bedrooms being very undesirable, particularly as the rooms are very small, and damage to the walls and woodwork results. The committee submitted a plan for the closing in of a portion of a large coal store so as to provide two sheds, one for the male, and the other for the female staff, accommodating a total number of 60 cycles, 30 for each sex. Authority was given by the Board for the alterations to be carried out at a cost of £90.

The children's committee in their report stated that they had received from Miss Jacob, matron of the East Cliff House, Margate, an application for an increase of salary, in view of the increased work and responsibility that had been thrown upon her since the opening of the new buildings, and it was decided to increase it from £65 per annum to £80, rising by annual increments of £5 to £90 per annum. The recommendation was adopted. The same committee further reported that they had had under consideration the appointment of a matron for the new ringworm schools at Banstead Road, Sutton, Surrey. They thought it desirable that a matron should be selected forthwith so that they might be in a position to obtain her advice on the questions of staff, equipment, and administration which were now under consideration. The committee unanimously gave notice of the following recommendation to be submitted at the next meeting of the Board, viz.:—That, subject to the approval of the Local Government Board, Miss Emily Turton be appointed matron of the Banstead Road School on the terms already approved, viz., £100 per annum, rising by annual increments of £5 to £150 per annum with board, lodging, and washing; and that Miss Turton be transferred from St. Anne's Home, Herne Bay, when the Banstead Road School is ready for opening by the managers.

### Everybody's Opinion.

[Correspondence on all subjects is invited, but we cannot in any way be responsible for the opinions expressed by our correspondents. No communication can be entertained if the name and address of the correspondent are not given as a guarantee of good faith, but not necessarily for publication. All correspondents should write on one side of the paper only.]

WOODLANDS CONVALESCENT HOME, RAWDON.

"The Matron" of this institution writes: Miss M. Walker has been appointed nurse and assistant to the matron, and not matron as stated in your issue of last week.

AN APPEAL FROM MEALSGATE, CARLISLE.

"Lady Gillford, President of the Bolton-Ireby and Uldale Branch of the Cumberland Nursing Association," writes: Will you allow me to state, with reference to an appeal from "Nurse Ireby" in "The Hospital" of February 1st, that the appeal does not come from the nurse employed by the Cumberland Nursing Association. I ask you to do this to remove any reflection of inefficiency as to the working of the District Nursing Association, which is supplied with the things asked for, and does, I am happy to say, what it can for the many subscribers in the village and neighbourhood.

CALLING PATIENTS BY THEIR NUMBER.

"An English Nurse Trained in New York" writes: I should like to know why the authorities in London hospitals refer to the poor patients by number. It seems to me needless cruelty, and would not be tolerated in the States. All patients in the best hospitals there are referred to as Miss A. or Mr. B. At the Belle Vue or General Hospital they are called by their Christian name. I think it is very lowering for a poor person to hear himself or herself called "Number So-and-so." Surely they could be called by the name they are entitled to with as little trouble as the number. I should just like to hear the views of other people on the matter.

### Novelties for Nurses.

INDIARUBBER GOODS.

By Our Shopping Correspondent.

If any of my readers are looking out for indiarubber goods, macintoshes, etc., they may like to know that Messrs. Anderson, Anderson and Anderson, of 37 Queen Victoria Street, London, E.C., are holding their annual stock-taking sale up to the 24th of this month. There is a variety of ladies' waterproofs at great reductions; these are in both cloak and coat shape, and in various shades. Some of them reduced to quite half the original price. There are some striped and plain cycling jackets, at very low prices; these are always useful, and can be worn for walking also if one takes the precaution to put on a skirt that will not spoil with rain.

Messrs. Anderson's water and air beds and cushions are almost too well known to need special attention drawn to them, but as this is an opportunity for getting them at a much lower price than usual I must give an idea of what is on sale. To begin with the largest: there are a few second-hand water-beds, full size, of which the original price was not much under £10. These are being offered for 35s. and upwards. Half-beds, 36 inches each way, are offered at 26s.; and there are varying sizes and prices between the two I have mentioned. Last season's water pillows and cushions are also very greatly reduced in price. Then there are air-pillows, plain, circular, and reeded; and nearly 100 waterproof sheets are offered, size 78 by 36 inches, with turned edges and eyeleted. These are reduced from 9s. 6d. to 5s. 11d., and a few may be had for 33s. 6d. There is also a very large assortment of remnants of waterproof sheeting, from which a selection might be made which would be useful to a district nurse. Other things to be seen at 37 Queen Victoria Street are nursing aprons, 10d. each, in black rubber; hot-water bottles; a pocket douche complete with 6-foot tubing, in a waterproof case; bed-pans, enemas, etc.; and I may add that anything can be had on approval if a postal order is sent, and that Messrs. Anderson offer to return the money if the goods are not kept.

### Appointments.

[No charge is made for announcements under this head, and we are always glad to receive, and publish, appointments. But it is essential that in all cases the school of training should be given.]

Basford District Infectious Hospital.—Miss Theresa Dickinson and Miss A. M. Caunt have been appointed nurses. Miss Dickinson was trained at Bradford Infirmary, and has since been sister at Keighley and Bingley Isolation Hospital. Miss Caunt was trained at Wisbeach General Hospital, where she has since been nurse.

Battle Workhouse.—Miss Phyllis Thompson has been appointed assistant nurse. She was trained by the Meath Workhouse Nursing Association at St. Peter's Home, Kilburn.

Bradford Royal Infirmary.—Miss E. E. Bell and Miss M. H. Pidgeon have been appointed sisters at the Bradford Royal Infirmary. Miss Bell was trained for three years at the Royal United Hospital, Bath, and for the past year has been charge nurse at the Grove Hospital, Tooting, S.W. Miss Pidgeon was trained at the Royal United Hospital, Bath, was for a year and a half sister in the same institution, and for the past year has been charge nurse at the Grove Hospital, Tooting, S.W. Miss Pidgeon has done private nursing, and holds the L.O.S. certificate.

Bridgewater Infirmary.—Miss Mary F. Way has been appointed matron. She was trained at St. Bartholomew's Hospital, London, and has since been lady superintendent of the Grosvenor Hospital for Women and Children, Westminster; sister of the surgical wards at University College Hospital, and matron *pro tem* of the East Suffolk and Ipswich Hospital. She has also done army nursing in South Africa for a year.

Brighton Union Infirmary.—Miss Lilian F. Carver and Miss Elizabeth Smith have been appointed nurses. Miss Carver was trained for three years at Shoreham Union Infirmary, where she was afterwards assistant nurse for one year. Miss Carver holds the L.O.S. certificate. Miss Smith was trained for three years at North London Hospital, Kentish Town Road, and was afterwards charge nurse for three years at City Hospital, Parkhill, Liverpool.

Burnley Union Infirmary.—Miss Annie Williams has been appointed charge nurse. She was trained, for three years, at Chorlton Union Hospital, West Didsbury, Manchester.

Camberwell Infirmary.—Miss Ellen E. Thomas has been appointed sister. She was trained at St. Mary's Hospital, Paddington, and was afterwards sister. She has since been temporary staff nurse at St. Peter's Hospital, W.C.

Cardiff Infirmary.—Miss K. Daniel has been appointed sister. She was trained at Cardiff Infirmary, where she has since been staff nurse, sister in male wards, and theatre sister.

Concentration Camps.—Miss A. M. Sharrock, who was lately appointed one of the nurses in the Orange River Concentration Camps, was trained at Springhill Infirmary, Birmingham, was afterwards sister there for three years, night sister at the Hahnemann Hospital, Liverpool, assistant matron at Crumpsall Infirmary, Manchester, and, for the last three years, superintendent nurse at Steyning Union Infirmary.

Fountain Fever Hospital, Tooting Grove.—Miss E. Adeleine Franklin and Miss Florence M. Bartleet have been appointed charge nurses. Miss Franklin was trained at St. George's Infirmary, Fulham Road, and has also been charge nurse at the Romford Union Infirmary, at St. George's Infirmary, and at the Grove Hospital, Tooting, S.W. Miss Bartleet has just finished four years' training at St. Thomas' Hospital.

Grantham Hospital.—Miss Esther C. Evans has been appointed charge nurse. She was trained at Burton-on-Trent Infirmary, where she has since been charge nurse. She has also done private nursing, and been temporary sister at the Women's Hospital, Sparkhill, Birmingham.

Hungerford Union Infirmary.—Miss Mary Anne Coffey has been appointed assistant nurse. She was trained by the Meath Workhouse Nursing Association at St. Peter's Home, Woking.

King's Norton Union Infirmary.—Miss Mary Elizabeth Cave, who has been appointed sister, was, after her training at Birmingham Infirmary, assistant nurse at Plymouth Union Infirmary for three years, private nurse at Herne Bay for some time, and for nearly three years charge nurse at Burnley Union Infirmary.

Kirkcaldy District Joint Hospital for Infectious Diseases.—Miss Annie Ellis has been appointed matron nurse. She was trained at Edinburgh Royal Infirmary, and was afterwards staff nurse at Chalmers Hospital, Edinburgh, and matron at Galashiels Cottage Hospital.

London School Board.—Miss R. Russell has been appointed lecturer on "Home Nursing" to the Lavender Hill and Brixton schools. She was trained at Westminster Hospital, and has since been Queen's nurse and private district nurse at Brixton.

Londonderry Union Infirmary.—Miss Florence Beattie and Miss Eleanor Walker have been appointed assistant nurses. Miss Beattie was trained at Dr. Steevens' Hospital, Dublin, and has since been private nurse in Winchester. She has also been staff nurse at Mercer's Hospital, Dublin, and for the last three years she has been on the staff of the Belfast Royal Hospital. Miss Walker was trained at the Belfast Royal Hospital.

Lytham Cottage Hospital.—Miss Barwick has been appointed matron. She was trained at the Leeds General Infirmary, and has since been sister at the Salop Infirmary, Shrewsbury, night superintendent at the Royal Infirmary Aberdeen, matron at Bridgend Cottage Hospital, and matron at the Pembrokeshire Infirmary.

Provincial Police Orphanage, Redhill, Surrey.—Miss Edyth M. Davis has been appointed matron. She was trained at West Ham Hospital, E., and has since been sister at Belfast Children's Hospital, superintendent nurse of Sheffield Children's Homes Hospital, and for the past two years matron of the Oriolet Hospital, Loughton, Essex.

Rodgett Infirmary, Royal Albert Asylum, Lancaster.—Miss Elizabeth Nichol has been appointed matron. She was trained for 3½ years at St. Mary's Hospital, Manchester, and was afterwards assistant nurse at Oldham Infirmary, private nurse on the staff of the Oldham Nursing Association, charge nurse, female medical block, and for some time maternity charge nurse at Fir Vale Infirmary, Sheffield, four years matron Falmouth Hospital and Dispensary, and 18 months sister-in-charge of a private nursing home in Manchester.

Union Hospital, Pontypridd.—Miss M. L. Mancer has been appointed superintendent nurse. She was trained at Shoreham Union Infirmary for three years, where she was afterwards assistant superintendent for twelve months She has since had charge of the isolation block at Whiston Infirmary, Prescot. Miss Mancer holds the L.O.S. certificate.

Victoria Cottage Hospital, Sidmouth.—Mrs. Irene Hancock has been appointed matron. She was trained at the Sussex County Hospital, Brighton, and has since been sister of the female medical and surgical ward at the Royal Portsmouth Hospital.

### An Off-Duty Letter.

### "ULYSSES." A POETIC PLAY.

Last Saturday I went to see "Ulysses" at Her Majesty's Theatre, and I think you will like to know my impressions. As to the story itself, Mr. Tree, mindful of the fact that many who will visit the theatre may have left their school days far behind them, and have but a hazy general idea of the theme of the play, presents to each person on arrival a tasteful little book, which tells in compact form the story of "Ulysses" as set forth in Homer's "Odyssey," together with the summary of that portion of the hero's life which is dealt with by Mr. Stephen Phillips, the author of the play. Therefore, it is wise to arrive early enough to master these details before darkness settles down over the theatre, and the curtain rises on Mount Olympus. Here, in the early dawn, Pallas Athené (Minerva) is pleading that Ulysses may be released from the spell woven around him by Calypso, and may return to his faithful wife, Penelope. Neptune opposes the request, claiming that the hero is his lawful prey; and finally Zeus only grants Athené's request upon condition that Ulysses shall win his way home through the terrors of Hades. The next scene is a sea cave on the charmed island of Ogygia, "an odorous, amorous isle of violets," where Ulysses has been content to remain for many years, desiring nothing better than to be encircled by the white clinging arms of the beautiful Calypso. Nymphs sing and dance around him on the sandy shore, and the air is filled with languorous music. Eventually Athené visits the hero in his sleep, and rouses once more his dead remembrance. Then, when he awakens, he is fired with a desire to return to Ithaca. Calypso seeks, by many tender wiles and some scorn, to turn him from his purpose; but neither the sketch she paints of Penelope with gray hair and dragging feet, nor her promise of immortality to Ulysses if he remain, avails. The ship is brought and manned, and with a last appeal from Calypso "Say farewell to me, but not to the thought of me," Ulysses sails away. The third scene is the forecourt of the Palace of Ulysses at Ithaca, in which high revels are being held. Wine and women and waste are all in evidence, and Telemachus, son of Ulysses, looks on with inert grief. Then Athené speaks to him, and tries to induce him to throw off his lethargy; but his assertion of authority has no result, and the three suitors of the Queen wax bolder and more insolent, and force Penelope to appoint the next full moon as the date of her decision.

The next act contains two scenes. One, "The Approach to Hades," the other, "The Interior." Without is the blackness of towering rocks against a lurid sky, whilst smoke from the burning torments within mounts ever slowly upwards, and the wails of "all the faint, unhappy host of hell" rise and fall on the heavy air. Small wonder that Ulysses turns back in horror, but ultimately his nobler self prevails, and, with Hermes as a guide, he enters the dreaded portal. As he stands at the top of a long flight of steps hewn out of the rocks, shadowy forms creep up and fly around him; at every movement some distraught soul asks him to aid, till at length he reaches the river Styx, where crouches Charon in his boat. At first the old man refuses to convey him over because flesh still hangs to his bones and blood runs in his veins. But on hearing that it is the decree of the gods, the boat moves across. There are interviews with Phædra and Teiresias; with Agamemnon, who recalls the way he was treated by his wife, and warns Ulysses accordingly; and with Anticleia, the mother of Ulysses, who, having but lately come to Hades, gives the welcome news that Penelope is still alive and faithful. Then the ordeal is over, and not all the outstretched hands of weeping phantoms can prevent the return of the hero to earth. This, though scenically the greatest triumph, is the act I like least. It is of course based upon the heathen idea of Hades, but, to my mind, it jars upon Christian feelings. The sobbing of the children, "babes who died young," as they beg Ulysses to take them back to where they can again gather flowers, is painful, and the idea of their innocence surrounded with such blackness, mental and physical, is far from pleasing. The spot is hardly "Hades" in the modern acceptation of the word, it is simply "Hell"; but as the ancients confounded the two, Mr. Phillips has of course a good case. As to the melancholy beauty, the weird splendour, of the whole, there can be no difference of opinion.

In the next scene, the shipwrecked mariner, having lost his boat and his crew, is thrown up on the coast of Ithaca. His guardian angel, Athené, cheers him on his way, he reveals himself to his old swineherd, Eumaeus, and to his son, and they arrange the manner in which the king shall claim his own. The finale is a fitting end to a striking piece. The day has come for Penelope's decision. The courtly throng, whilst awaiting the advent of the queen, bait the poor beggar—in whose guise Ulysses is concealed—and before her decision, at the beggar's suggestion, each suitor tries to bend Ulysses' bow. None succeed save the rightful owner, who in doing so reveals himself, and as the suitors and their servants—save such as lay slain upon the floor—depart, the husband and wife meet in one long embrace.

As to the play itself, which is now published in book form, it is impossible at a single hearing to appreciate the grace and glow of the words. It may be wanting in humour, but for beauty of thought expressed in poetic phrase; for rich melody ringing out with majestic measure from well-chosen words; for the successful portrayal of the heights of love and the depths of despair, it is long since most of us have enjoyed such an intellectual treat. As to the acting, it seems a little hard that the man upon whom falls the burden of the principal representation should not win our highest praise. But that is the fault of the character, rather than of the actor. It is difficult to feel enthusiastic about a man who for more than a decade forgets his wife; who is spoken of as the prince of liars; who is exceedingly human in his cowardice; and who, but for the good offices of the gods, would never have achieved his goal. But in Mr. Tree's hands the so-called "hero" is rendered as attractive as possible. Penelope, in the person of Miss Lily Hanbury, is as lovely and as regal as even Homer could have wished. But the chief honours fall to Miss Constance Collier. She holds herself like a goddess, her statuesque beauty is most impressive, and her declamation is superb. She speaks as if she had lisped in poetry even in her early days; the measured rhythm never seems to fall from unaccustomed lips, and every word is clear and intelligible. Miss Nancy Price, as Calypso, is sufficiently seductive and bewitching to suggest an excuse for the lengthened absence of Ulysses, and her sudden changes from anger to love are cleverly conceived. Mr. Gerald Lawrence has little to do as Telemachus, but does it well, and the same may be said of Mr. Oscar Ashe as Antinous, and Miss Winifred Arthur-Jones as Venus. The humour of the piece, which is slight, is admirably sustained by Mr. Henry Kemble as the suitor Ctesippus, and Mr. Lionel Brough as the swineherd Eumaeus. Mention, too, must be made of Mr. Courtice Pounds, the sweet-voiced minstrel who interprets vocally Mr. Coleridge Taylor's delightful music. This also includes an interesting prelude, some graceful and suggestive choruses, and much ingenious orchestration—a distinct aid to the success of the representation.

### For Reading to the Sick.

THE SEASON OF LENT.

THE message of the Lenten season is, "Repent ye, for the Kingdom of Heaven is at hand." "If we confess our sins, He is faithful and just to forgive us our sins, and to cleanse us from all unrighteousness." Let us do our part, and leave the rest to Jesus.—*H. J. Wilmot Buxton.*

I need Thy presence every passing hour.
What but Thy grace can foil the tempter's power?
Who like Thyself my guide and stay can be?
Through cloud and sunshine, Lord, abide with me.

*Lyte.*

If on our daily course our mind
Be set to hallow all we find,
New treasures still, of countless price,
God will provide for sacrifice.

The trivial round, the common task,
Will furnish all we need to ask.
Room to deny ourselves, a road
To bring us daily nearer God.

Only, O Lord, in Thy dear love
Fit us for perfect rest above:
And help us, this and every day,
To live more nearly as we pray.

*Keble.*

The holy, the pure-hearted, and the penitent have fellowship with angels and walk with God, and God dwells in them with a growing nearness day by day: they are ever more and more one with Him, and partake more fully of the Divine nature and are filled with the will of God: they abide in God, and God in them: they are one with Christ and Christ with them: they are taken up as it were into the company of heaven, and, by the ascent of their moral being, climb upwards to the throne of God.

*H. E. M.*

This is the great business and meaning of our life on earth: that we should more and more yield up our hearts to God's great grace of love: that we should let it enter ever more fully and more freely into us, so that it may even fill our whole heart and life. We must day after day be driving back, in His strength, the sin that doth so easily beset us, and the selfishness that sin has fastened in our hearts: and then His love will day by day increase in us. Prayer will win and keep it; work will strengthen and exercise it; the Bible will teach us how to know and prize it, how to praise God for it; the Holy Eucharist will ever renew and quicken its power in our hearts. And so (blessed be God!) love and joy and peace will grow in us, beyond all that we can ask or think; and He will forgive us, for love's sake, all the failures, all the faults in whatever work He has given us to do; and will bring us at last into the fullness of that life which even here He has suffered us to know; into that one Eternal Home where Love is perfect and unwearied and unending, and where nothing ever can part us from one another or from Him.—*Dean Paget.*

### Notes and Queries.

**The Editor is always willing to answer in this column, without any fee, all reasonable questions, as soon as possible.**
**But the following rules must be carefully observed:—**
**1. Every communication must be accompanied by the name and address of the writer.**
**2. The question must always bear upon nursing, directly or indirectly.**
**If an answer is required by letter a fee of half-a-crown must be enclosed with the note containing the inquiry, and we cannot undertake to forward letters addressed to correspondents making inquiries. It is therefore requested that our readers will not enclose either a stamp or a stamped envelope.**

*Lip-reading.*

(180) If A. M. lives in the Midlands or North of England, I advise her to write to Miss Crossley, St. Thomas's Vicarage, Batley, Yorks, for information about lip-reading.—*E. P.*

*Swansea.*

(181) Is there an eye hospital in Swansea? Is it a large one, and if not, is there a special department for eyes at the General Hospital there?—*Nurse E. E. C.*

The name of the hospital at Swansea is the "Swansea General and Eye Hospital," and there are two ophthalmic surgeons on the staff.

*Mourning.*

(182) Will you kindly tell me what is the usual way for a trained nurse to wear mourning, her outdoor uniform being navy blue, and her indoor white?—*F. M. W.*

It is unnecessary to wear mourning when in uniform, but if you wish to do so place a band of black round your left arm.

*Disinfecting.*

(183) 1. What is the best way of disinfecting children after measles; is washing with carbolic soap sufficient? 2. What is the cheapest way of buying carbolic for disinfecting linen, etc.?—*Perplexed.*

A good bath with plenty of soap and water, and entirely fresh clothing, should answer the purpose. 2. Messrs. Calvert's preparations are cheap and good.

*Hamburg.*

(184) Will you let me know if there are any private English nursing homes in Germany—Hamburg for instance. Would it be difficult for a nurse, comparatively ignorant of the language, to get on there?—*Beth.*

Write for information to the English Consul of the place to which you wish to go. Remuneration is frequently very small, private institutions sometimes only charging 16s. to 20s. a week for their nurses.

*Maternity.*

(185) I was engaged to nurse a lady from August 21st. On August 4th I was called in, but my services not being required, I waited through August and September. I had then to go to town for a few days on business and found on my return that another nurse had been called in in my absence, although the baby was not born until two hours after my return. I had neglected to leave my address, does that make any difference? Can I claim half fees for the time I was kept waiting?—*Nurse E.*

You can claim half fees from August 21st to the date of the child's birth. If another nurse had to be called in on account of your having omitted to leave an address, it would be only fair for you to compensate her for the time she fulfilled your duties; that is supposing you resumed charge of the case on your return.

Will you kindly tell me of an institution where maternity nurses without general training are received?—*Nurse B.*

You must endeavour to obtain what you want through advertisements.

*Stewardess.*

(186) Will any reader of THE HOSPITAL tell a nurse how she may obtain a situation as stewardess in a good line of steamers?—*A. E. S.*

Apply to the secretaries of the various shipping companies.

**Standard Books of Reference.**

"The Nursing Profession: How and Where to Train." 2s. net; post free 2s. 4d.
"Burdett's Official Nursing Directory." 3s. net; post free, 3s. 4d.
"Burdett's Hospitals and Charities." 5s.
"The Nurses' Dictionary of Medical Terms." 2s.
"Burdett's Series of Nursing Text-Books." 1s. each.
"A Handbook for Nurses." (Illustrated). 5s.
"Nursing: Its Theory and Practice." New Edition. 3s. 6d.
"Helps in Sickness and to Health." Fifteenth Thousand. 5s.
"The Physiological Feeding of Infants." 1s.
"The Physiological Nursery Chart." 1s.; post free, 1s. 3d.
"Hospital Expenditure: The Commissariat." 2s. 6d.
All these are published by the SCIENTIFIC PRESS, Ltd., and may be obtained through any bookseller or direct from the publishers 28 and 29 Southampton Street, London, W.C.

